# Long Non-coding RNA CDKN2B Antisense RNA 1 Gene Contributes to Paclitaxel Resistance in Endometrial Carcinoma

**DOI:** 10.3389/fonc.2019.00027

**Published:** 2019-01-29

**Authors:** Chao Shang, Cheng N. Ao, Chi C. Cheong, Lirong Meng

**Affiliations:** ^1^Department of Neurobiology, School of Life Science, China Medical University, Shenyang, China; ^2^School of Health Sciences, Macao Polytechnic Institute, Macau, China

**Keywords:** non-coding RNA, endometrial carcinoma, CDKN2B antisense RNA 1, miR-125a-5p, chemotherapy, paclitaxel

## Abstract

Endometrial cancer (EC) is the most common malignancy of the female reproductive tract. In this study, we clarified the clinical significance of CDKN2B antisense RNA 1 (CDKN2B-AS) gene, and its effects on paclitaxel sensitivity in EC. Firstly, CDKN2B-AS gene was highly expressed in EC tissues and cell lines. The high-expression of CDKN2B-AS gene was associated with high pathological grade and low paclitaxel sensitivity of EC tissues. Knockdown of CDKN2B-AS gene sensitized Ishikawa/PA and HEC1A/PA cells to paclitaxel, and promoted paclitaxel-induced cytotoxicity. Secondly, the low-expression of miR-125a-5p was closely associated with low paclitaxel sensitivity of EC cells, and up-regulation of miR-125a-5p could increase paclitaxel sensitivity of Ishikawa/PA and HEC1A/PA cells. MiR-125a-5p also mediated the suppressive effects of knockdown of CDKN2B-AS on paclitaxel resistance in EC cells. Thirdly, B-cell lymphoma-2 (Bcl2) and Multidrug Resistance-Associated Protein 4 (MRP4) genes were target genes of miR-125a-5p, which modulated paclitaxel resistance of Ishikawa/PA and HEC1A/PA cells through targeted silencing Bcl2 and MRP4. In conclusion, high-expression of CDKN2B-AS is associated with a poor response to paclitaxel of EC patients, and knockdown of CDKN2B-AS inhibits paclitaxel resistance through miR-125a-5p-Bcl2/MRP4 pathway in EC patients. Our findings help elucidate the molecular mechanisms of chemoresistance in EC patients.

## Introduction

Endometrial cancer (EC) is one of the most common malignancy of the female reproductive tract and is increasing in incidence. The mortality of EC is next to ovarian and cervical cancer, and that is currently increasing year-by-year (1, 2). Chemotherapy is extensively used for treatment of EC, and it can significantly improve the prognosis and inhibit the recurrence and metastasis (3). Drug resistance reduces the sensitivity to chemotherapeutic drugs, and contributes a barrier, leading to treatment failure of EC. Accordingly, that is pivotal to identify the therapeutic target, re-sensitizing EC to chemotherapeutic drugs and its underlying mechanism. Several scholars have proposed diverse hypotheses, accounting for chemotherapy failure of EC patients, including DNA repair deregulation, aberrant function of efflux pumps, imbalance of signaling pathway, and so on Brasseur et al. (4), Shang et al. (5), and Liu et al. (6).

In recent years, non-coding RNAs (ncRNAs) have become a hotspot in the research of life science, especially in oncology, involving long non-coding RNAs (lncRNAs) and short non-coding RNA (e.g., microRNAs). Recent studies reported that some ncRNAs could be prognostic and diagnostic biomarkers, as well as being therapeutic targets for tumors (7–9). Increasing evidences have indicated that ncRNAs are involved in formation and progress of chemotherapy resistance, including EC (10–12). Our previous study revealed that Tumor Suppressor Candidate 7 (TUSC7) gene could specifically combine and silence miR-23b, and then inhibit the resistance of cisplatin and taxol in EC (12). Our preliminary experiments showed that CDKN2B antisense RNA 1 (CDKN2B-AS) gene was associated with paclitaxel resistance of EC, thus, we attempted to study the molecular mechanism of CDKN2B-AS triggered regulation for chemotherapy resistance of EC.

CDKN2B antisense RNA 1 (CDKN2B-AS) gene is a lncRNA gene identified by Pasmant et al. during the genetic study of a melanoma-neural system tumor family in 2007, that also named Antisense Non-coding RNA In The INK4 Locus (ANRIL) (13). Recent studies revealed that the CDKN2B-AS gene was up-regulated and acted as an oncogene in several malignant tumors, such as breast cancer, prostate cancer, and cervical cancer (14–17). At present, there is no report on CDKN2B-AS gene associated with EC. It is well-known that lncRNA could be associated with microRNA to regulate its expression and function. This study demonstrated that CDKN2B-AS could specifically silence miR-125a-5p expression of in EC cells.

MiR-125a-5p originates from 5′ end of pre-miR-125a and belongs to miR-125 family. Accumulating evidences found that miR-125a-5p was down-regulated in a variety of tumors and participated in the tumorigenesis and malignant progression of tumors by regulating its target genes to play the role of a tumor suppressor gene (18–20). The expression and potential function of miR-125a-5p in EC patients have still remained unclear. Our findings confirmed that B-cell lymphoma-2 (Bcl2) and Multidrug Resistance-Associated Protein 4 (MRP4) were the target genes of miR-125a-5p.

It is widely accepted that B-cell lymphoma-2 (Bcl2) gene is an important member of Bcl2 family and can inhibit apoptosis and promote cell survival, while Bcl2 has abnormal expression or function in almost all tumors. Defect to the Bcl-2 gene has been identified as a cause of resistance to cancer treatments. Bcl2 was negatively regulated by lncRNA GAS5 and contributed to doxorubicin resistance of bladder transitional cell carcinoma (21). Notch3-specific inhibition reduced the expression of Bcl2 and reversed paclitaxel resistance of ovarian cancer (22). Chon et al. reported that knockdown of Bcl2 antagonist of cell death (BAD) pathway increased the cisplatin resistance of Ishikawa and HEC1-A cells (23). However, it is not clear enough whether Bcl2 is involved in paclitaxel resistance of EC.

MRP4 gene belongs to ATP-binding cassette (ABC) transporters superfamily, that also named ATP Binding Cassette Subfamily C Member 4 (ABCC4). MRP4 can expel the chemotherapeutic drugs from the cells before they work, so as to reduce the damage of the chemotherapeutic drugs to the cells. (24). Overexpression of MRP4 mediated the acquired docetaxel resistance, targeting MRP4 treatment which re-sensitized docetaxel-resistant prostate cancer cells to docetaxel chemotherapy (25). The roles of MRP4 on chemotherapy resistance of EC need to be further studied.

Therefore, this study explored the clinical significance of CDKN2B-AS expression, and clarified the mechanism of CDKN2B-AS, contributing to paclitaxel resistance through miR-125a-5p/Bcl2&MRP4 pathway in EC.

## Materials and Methods

### Clinical Specimens

In this study, 87 cases of EC patients were diagnosed and treated at Shengjing Hospital of China Medical University (Shenyang, China) from October 2015 to November 2016. The paracancerous normal endometrium tissue (PNET) and EC tissue specimens were obtained through hysteroscopy accompanied with biopsy, and diagnosed by two pathologists, in which the complete clinical data were collected as well. All the patients were not treated with radiotherapy or chemotherapy before diagnosis. The EC tissues specimens without treatment were used to detect the expression level of CDKN2B-AS and miR-125a-5p in EC patients. After diagnosis, all EC patients were treated with paclitaxel. After two or three cycles of chemotherapy, the curative effects were verified according to hysteroscopy with biopsy and imaging detection, and all EC patients were divided into two groups, including sensitive group (*n* = 36) and insensitive group (*n* = 51).

This study was conducted in accordance with the Declaration of Helsinki, and was approved by the Ethics Committee of Shengjing Hospital of China Medical University, and written informed consent was obtained from all participants as well.

### Cell Lines and Culture

Human endometrial cell lines (HEC-251), human EC cell lines (Ishikawa, HEC-1A), and human embryonic kidney cell lines (HEK293T) were obtained from the Cell Resource Center of Chinese Academy of Medical Sciences (Beijing, China). Paclitaxel-resistant EC cell lines (Ishikawa/PA and HEC1A/PA cell lines) were set up previously from parental cell lines (Ishikawa, HEC-1A), and stored in our laboratory (12). Those cells were cultured in Dulbecco's modified Eagle's medium (DMEM), containing 10% fetal bovine serum (FBS; Shanghai ExCell Biology, Inc., Shanghai, China) in a 95% air/5% CO_2_ incubator at 37°C.

### Quantitative Reverse Transcription Polymerase Chain Reaction (RT-qPCR)

Total RNA was extracted using TRNzol reagent (TIANGEN, Beijing, China) and reversely transcribed into cDNA using lnRcute lncRNA First-Strand cDNA Synthesis Kit (TIANGEN, Beijing, China). The expression level of CDKN2B-AS was examined using an lnRcute lncRNA qPCR Detection Kit (TIANGEN, Beijing, China) in accordance with manufacturer's instructions. The sense primer of CDKN2B-AS was 5′-TGCTCTATCCGCCAATCAGG-3′ and its antisense primer was 5′-GGGCCTCAGTGGCACATACC-3′ (26), in which the specificity was checked, that could not be used to amplify CDKN2B gene. The expression level of miR-125a-5p was examined with Taqman Universal Master Mix II (Life Technologies, Carlsbad, CA, USA). The relative expression levels of CDKN2B-AS and miR-125a-5p were calculated using 2^−ΔΔCT^ method after normalization with reference genes (β-actin and U6).

### Cells Transfection

The inhibitor of CDKN2B-AS (smart silencer-CDKN2B-AS, ss-CDKN2B-AS) and its negative control (ss-NC) were designed and synthesized by Ribobio Co. (Guangzhou, China), and transfected into EC cells via HiPerFect reagent (QIAGEN, Hilden, Nordrhein-Westfalen, Germany) in a 6-well-culture plate in accordance with the manufacturer's instructions. The stable transfected cells were selected using Geneticin (Sigma-Aldrich, St Louis, MO, USA).

The agonist and antagonist of miR-125a-5p (agomiR-125a-5p and antagomiR-125a-5p), as well as their negative controls (agomiR-NC and antagomiR-NC) were synthesized by GenePharma Co. Ltd. (Shanghai, China). The expression plasmid of Bcl2 and MRP4 (pUC-Bcl2 and pUC-MRP4) and their negative control (pUC-NC) were synthesized by Cyagen Inc. (Santa Clara, CA, USA). The microRNAs and plasmids were transiently transfected into EC cells using HiPerFect reagent.

### Cell Proliferation Assay

Enhanced Cell Counting Kit-8 (Beyotime Institute of Biotechnology, Beijing, China) was applied to examine cell proliferation. The cells in logarithmic growth phase were digested with trypsin, washed by phosphate-buffered saline (PBS), and suspended in the culture medium. Then, 2,000 cells in 100 μl medium were added into one pore of 96-well plates, 10 μl enhanced CCK-8 solution was added, and incubated for 1 h. The value of optical density was detected with the help of an MK3 microplate reader (Thermo Fisher Scientific, Waltham, MA, USA) at the wavelength of 450 nm.

### Cell Apoptosis Detection

Annexin V-FITC/PI Apoptosis Detection Kit (Jiancheng, Nanjing, Jiangsu, China) was used to detect cell apoptosis rate according to the manufacturer's instructions. In addition, 2 × 10^5^ cells were re-suspended in 500 μl binding buffer, 5 μl Annexin V-FITC and 5 μl Propidium iodide (PI) were added, and incubated at 25°C for 10 min. The apoptosis rate was detected and analyzed by FACScan flow cytometry with Diva 8.0 software (Becton Dickinson, Franklin Lakes, NJ, USA). The apoptosis rate was presented as the percentage of cells with FITC-Annexin V positive/PI negative in the right lower quadrant.

### Drug Sensitivity Assay

The Ishikawa/PA and HEC1A/PA cells were treated with paclitaxel (10, 20, 50, 100, and 150 mg/L) (12). The cell viability was examined after 24 h. Then, the half maximal inhibitory concentration (IC50) of paclitaxel was calculated according to their dose-response curve.

### Western Blotting

Protein of cells was extracted using a Protein Extraction Kit (Beyotime Institute of Biotechnology, Beijing, China), and quantified by using a Bradford Protein Assay Kit (Beyotime Institute of Biotechnology, Beijing, China). Protein (30 μg) was separated by polyacrylamide gel electrophoresis and transferred to a polyvinylidene fluoride (PVDF) membrane. PVDF membrane was blocked with Tween-Tris-buffered saline (TTBS), containing 5% non-fat milk at 25°C for 2 h, and hybridized with Bcl2 and MRP4 antibodies (#2872 and #12705; Cell Signaling Technology, Danvers, MA, USA) at 4°C overnight. After that, PVDF membrane was incubated with the second antibody at 25°C for 2 h. The PVDF membrane was treated with Chemiluminescent reagent (TIANGEN, Beijing, China) to visualize the bands. Then, the bands were analyzed by ImageJ software (NIH, Bethesda, MD, USA).

### RNA Pull-Down Assay

The biotinylated probes for CDKN2B-AS and miR-125a-5p (bio-CDKN2B-AS-W and bio-miR-125a-5p-W, containing wild-type binding site), as well as their negative controls (bio-CDKN2B-AS-M and bio-miR-125a-5p-M, containing mutant binding site) were synthetized by GenePharma Co. Ltd. (Shanghai, China). Probes were dissolved in the buffer and incubated with Dynabeads M-280 Streptavidin (Thermo Fisher Scientific, Waltham, MA, USA) for 10 min at 25°C to form probe-coated beads. Those probe-coated beads were incubated with the lysates from Ishikawa/PA and HEC1A/PA cells, and eluted with the washing buffer. The pulled down RNAs were detected by RT-qPCR.

### RNA Immunoprecipitation (RIP) Assay

RIP was assayed using a Magna RIP™ RNA-Binding Protein Immunoprecipitation Kit (Millipore Sigma, Burlington, MA, USA) according to the manufacturer's instructions and a previous study (27). Whole-cell lysate from Ishikawa/PA and HEC1A/PA cells was incubated with RIP buffer, containing magnetic beads conjugated with human anti-Ago2 antibody or negative control normal mouse IgG. Samples were incubated with Proteinase K, and immunoprecipitated RNA was isolated. The RNA concentration was measured by a spectrophotometer (NanoDrop, Thermo Fisher Scientific, Waltham, MA, USA) and the quality of RNA was assessed by using a bio-analyzer (Agilent, Santa Clara, CA, USA). Furthermore, purified RNAs were extracted and analyzed by RT-qPCR to demonstrate the presence of the binding targets.

### Luciferase Reporter Assay

The luciferase reporter plasmids (Bcl2-W and MRP4-W, containing wild-type binding site; Bcl2-M and MRP4-M, containing mutant binding site) were synthesized by the GenScript Co. (Piscataway, NJ, USA). HEK293T cells were co-transfected with the luciferase reporter plasmids and microRNAs, respectively. The Luciferase Reporter Kit (Beyotime Institute of Biotechnology, Beijing, China) was applied to detect the luciferase activity after 48 h in accordance with manufacturer's instructions.

### Statistical Analysis

Each experiment was repeated five times, the data were presented as mean ± standard deviation (SD), and analyzed with Student's *t*-test and one-way analysis of variance (ANOVA) using SPSS 22.0 software (IBM, Armonk, NY, USA). If the *P* < 0.05, the difference was statistically significant.

## Results

### The Expression Level of CDKN2B-AS Was Up-Regulated in EC

**Figure 1:**
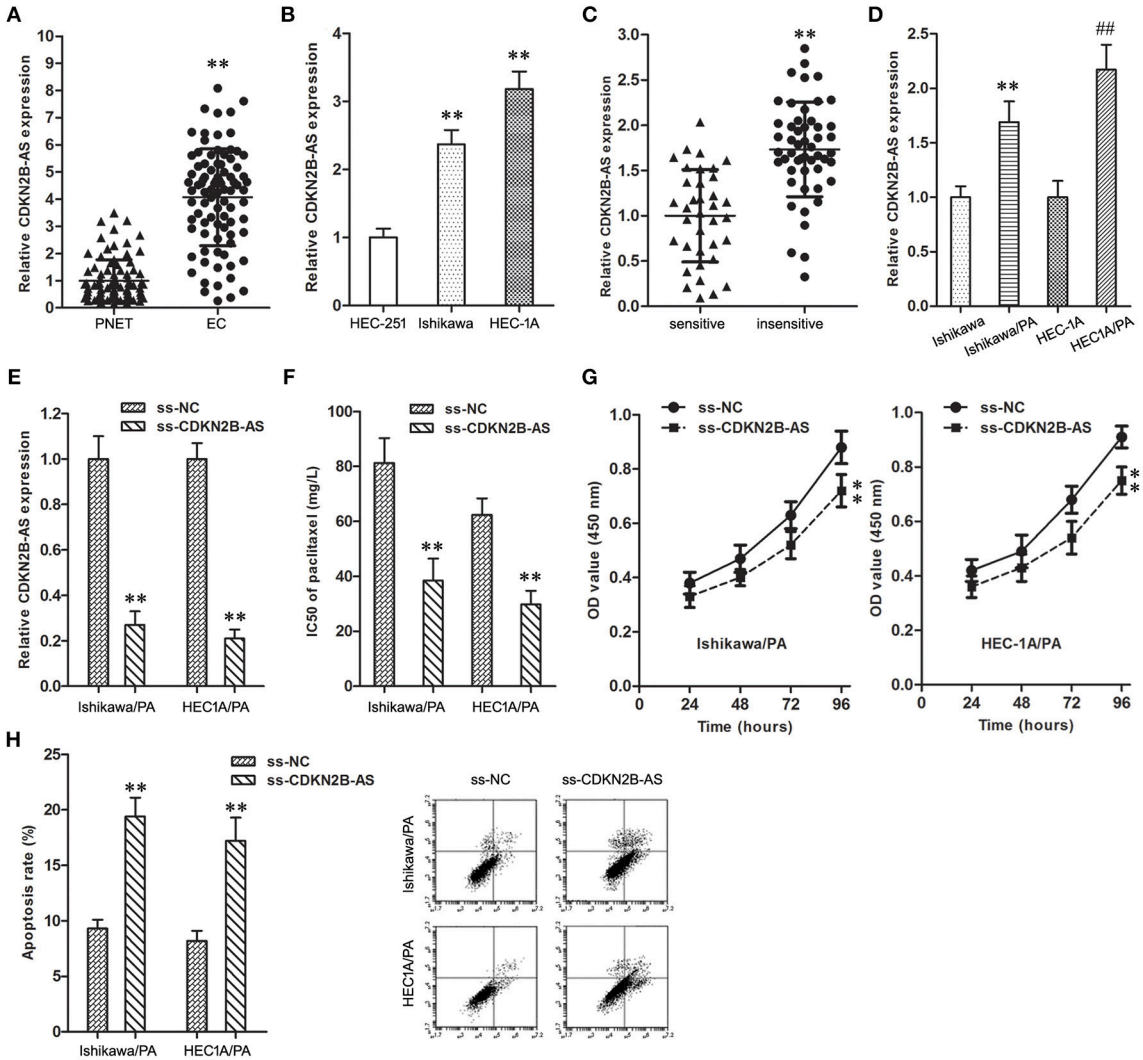
High-expression of CDKN2B-AS was correlated with poor response to paclitaxel in EC**. (A)** The expression of CDKN2B-AS gene in PNET and EC specimens. **P < 0.01 vs. PNET group. **(B)** The expression of CDKN2B-AS gene in HEC-251, Ishikawa and HEC-1A cells. **P < 0.01 vs. HEC-251 group. **(C)** The expression of CDKN2B-AS gene in paclitaxel sensitive and insensitive EC specimens. **P < 0.01 vs. sensitive group. **(D)** The expression of CDKN2B-AS gene in Ishikawa, Ishikawa/PA, HEC1A and HEC-1A/PA cells. **P < 0.01 vs. Ishikawa group; ##*P* < 0.01 vs. HEC1A group. **(E)** The expression of CDKN2B-AS gene in Ishikawa/PA and HEC-1A/PA cells. **P < 0.01 vs. ss-NC group. **(F)** The IC50 of paclitaxel in Ishikawa/PA and HEC-1A/PA cells. **P < 0.01 vs. ss-NC group. **(G)** The cell viability of Ishikawa/PA and HEC-1A/PA cells under treating with 10 mg/L paclitaxel. **P < 0.01 vs. ss-NC group. **(H)** The cell apoptosis of Ishikawa/PA and HEC-1A/PA cells under treating with 10 mg/L paclitaxel. **P < 0.01 vs. ss-NC group.

CDKN2B-AS gene in EC specimens was up-regulated in comparison with matched PNET specimens (Figure 1A). In addition, the expression level of CDKN2B-AS gene in Ishikawa and HEC-1A cells was significantly higher than that in HEC-251 cells (Figure 1B).

As shown in Table 1, the high-expression of CDKN2B-AS showed a positive correlation with high pathological grade of EC, however, that was not correlated with other clinical characteristics, including patient's age, estrogen receptor, and progesterone receptor. These findings suggested that CDKN2B-AS gene was involved in the progress of EC.

**Table 1:** The correlation analysis between the expression of ANRIL gene and the clinicopathological characteristics of 87 EC tissues.

**Factors**		**Case**	**Relative expression of CDKN2B-AS**	***P***
Age(years)	>54	43	4.01 ± 1.23	0.257
	≤54	44	4.13 ± 1.38	
Pathological grading	I–II	55	3.81 ± 1.09	0.011^*^
	III	32	4.52 ± 1.42	
Estrogen receptor	Negative	39	3.85 ± 1.36	0.195
	Positive	48	4.25 ± 1.48	
Progesterone receptor	Negative	41	3.78 ± 1.22	0.056
	Positive	46	4.33 ± 1.41	
Chemotherapy	Sensitive	36	2.84 ± 1.43	<0.001^**^
	Insensitive	51	4.92 ± 1.47	

* *P < 0.05*,

** *P < 0.01*.

### High-Expression of CDKN2B-AS Was Correlated With Poor Response to Paclitaxel in EC

Furthermore, the over-expression of CDKN2B-AS was associated with the low paclitaxel sensitivity of EC patients (Table 1 and Figure 1C), and the expression level of CDKN2B-AS in Ishikawa/PA and HEC1A/PA cells was significantly higher than that in Ishikawa and HEC1A cells (Figure 1D), which preliminarily confirmed that CDKN2B-AS participated in the genesis of chemotherapy resistance in EC cells.

To verify the roles of CDKN2B-AS on chemotherapy resistance, the expression level of CDKN2B-AS was knockdown in Ishikawa/PA and HEC1A/PA cells to carry out loss-of-function assays (Figure 1E). Knockdown of CDKN2B-AS decreased the IC50 of paclitaxel from 81.29 and 62.37 mg/L to 38.41 and 29.74 mg/L in Ishikawa/PA and HEC-1A/PA cells, respectively, (Figure 1F), which certified that knockdown of CDKN2B-AS sensitized Ishikawa/PA and HEC-1A/PA cells to paclitaxel. Moreover, under treating with paclitaxel (10 mg/L), knockdown of CDKN2B-AS depressed cell viability (Figure 1G) and promoted apoptosis in Ishikawa/PA and HEC-1A/PA cells (Figure 1H). Besides, knockdown of CDKN2B-AS gene significantly increased paclitaxel-induced cytotoxicity.

### CDKN2B-AS Silenced the Expression Level of miR-125a-5p in EC Cells

**Figure 2:**
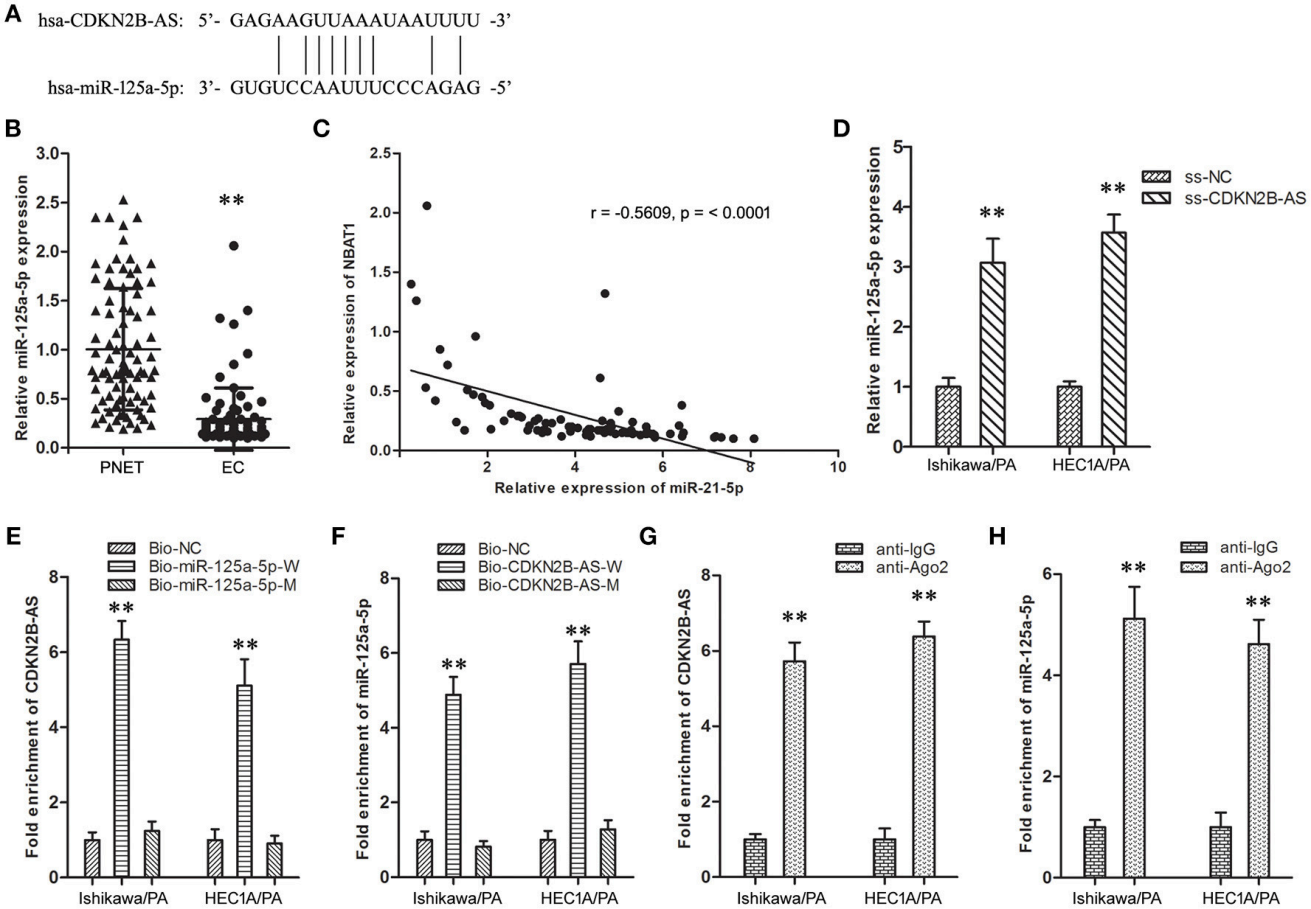
CDKN2B-AS silenced specifically miR-125a-5p expression of in EC cells**. (A)** The predicted miR-125a-5p binding site in the CDKN2B-AS sequence. Short vertical lines indicated complementary nucleotides. **(B)** The expression of CDKN2B-AS gene in PNET and EC specimens. **P < 0.01 vs. PNET group. **(C)** The co-expression patterns analysis between CDKN2B-AS and miR-125a-5p in EC. **(D)** The expression of miR-125a-5p gene in Ishikawa/PA and HEC-1A/PA cells. **P < 0.01 vs. ss-NC group. **(E)** Detection of CDKN2B-AS using qRT-PCR in the sample pulled down by biotinylated miR-125a-5p. **P < 0.01 vs. Bio-NC group. **(F)** Detection of miR-125a-5p using qRT-PCR in the sample pulled down by biotinylated CDKN2B-AS. **P < 0.01 vs. Bio-NC group. **(G)** Detection of CDKN2B-AS using qRT-PCR in RNA immunoprecipitation complex. **P < 0.01 vs. anti-IgG group. **(H)** Detection of miR-125a-5p using qRT-PCR in RNA immunoprecipitation complex. **P < 0.01 vs. anti-IgG group.

The online databases (TargetScan 7.1 and Starbase 2.0) predicted a specific combination between CDKN2B-AS and miR-125a-5p (Figure 2A). Then, the expression level of miR-125a-5p in EC specimens was down-regulated in comparison with matched PNET specimens (Figure 2B), and analysis of the co-expression patterns showed a negative correlation between CDKN2B-AS and miR-125a-5p in EC cells (Figure 2C, *r* = −0.5609, *P* < 0.001). Knockdown of CDKN2B-AS significantly up-regulated the expression level of miR-125a-5p in Ishikawa/PA and HEC-1A/PA cells (Figure 2D). In addition, RNA pull-down assay identified CDKN2B-AS could be combined with bio-miR-125a-5p-W probe, except for bio-miR-125a-5p-M (Figure 2E). Similarly, miR-125a-5p could be combined with bio-CDKN2B-AS-W probe, while that was not observed for bio-CDKN2B-AS-M (Figure 2F). Furthermore, RIP experiments confirmed that CDKN2B-AS and miR-125a-5p were both enriched in anti-Ago2 group (Figures 2G,H), and they were in a RNA-induced silencing complex (RISC). These results indicated that CDKN2B-AS and miR-125a-5p were associated with Ago-2 protein in a RISC complex, and CDKN2B-AS decreased the expression level of miR-125a-5p in a RISC-dependent manner, which was a classical regulatory manner of lncRNAs, regulating microRNAs.

### Up-Regulation of the Expression Level of miR-125a-5p Inhibited Paclitaxel Resistance in EC

**Figure 3:**
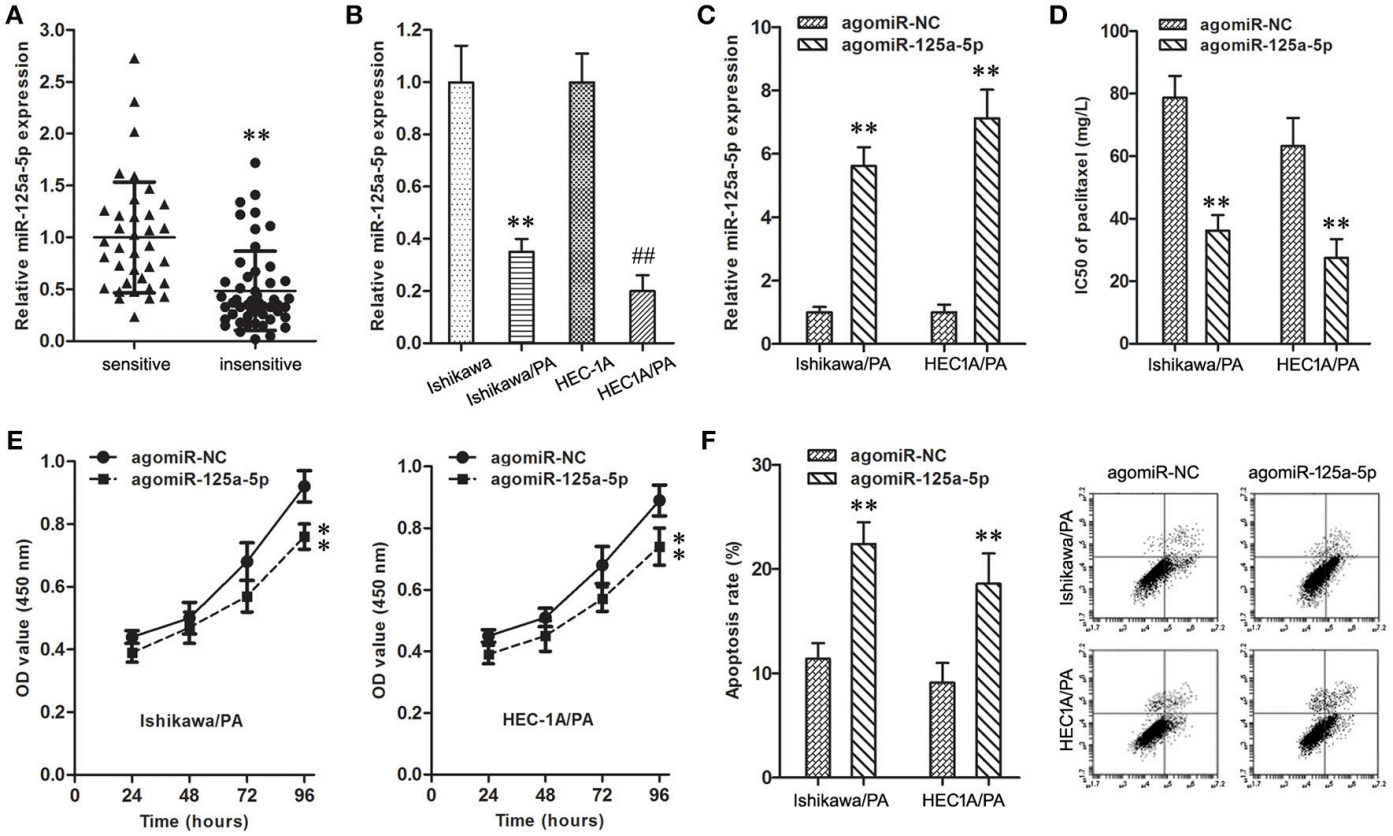
Up-regulation of miR-125a-5p inhibited paclitaxel resistance in EC**. (A)** The expression of miR-125a-5p gene in paclitaxel sensitive and insensitive EC specimens. **P < 0.01 vs. sensitive group. **(B)** The expression of miR-125a-5p gene in Ishikawa, Ishikawa/PA, HEC1A and HEC-1A/PA cells. **P < 0.01 vs. Ishikawa group; ##*P* < 0.01 vs. HEC1A group. **(C)** The expression of miR-125a-5p gene in Ishikawa/PA and HEC-1A/PA cells. **P < 0.01 vs. agomiR-NC group. **(D)** The IC50 of paclitaxel in Ishikawa/PA and HEC-1A/PA cells. **P < 0.01 agomiR-NC group. **(E)** The cell viability of Ishikawa/PA and HEC-1A/PA cells under treating with 10 mg/L paclitaxel. **P < 0.01 vs. agomiR-NC group. **(F)** The cell apoptosis of Ishikawa/PA and HEC-1A/PA cells under treating with 10 mg/L paclitaxel. **P < 0.01 vs. agomiR-NC group.

The expression level of miR-125a-5p in resistant EC patients was down-regulated in comparison with sensitive patients (Figure 3A). Similarly, compared with Ishikawa and HEC1A cells, the expression level of miR-125a-5p was also down-regulated in Ishikawa/PA and HEC1A/PA cells (Figure 3B).

To identify the role of miR-125a-5p on paclitaxel resistance, the expression level of miR-125a-5p was up-regulated in Ishikawa/PA and HEC1A/PA cells to carry out gain-of-function assays (Figure 3C). Additionally, up-regulation of miR-125a-5p decreased the IC50 of paclitaxel from 78.68 to 63.24 mg/L to 36.17 and 27.52 mg/L in Ishikawa/PA and HEC-1A/PA cells, respectively, (Figure 3D), which certified that up-regulation of miR-125a-5p sensitized Ishikawa/PA and HEC-1A/PA cells to paclitaxel. Moreover, under treatment with paclitaxel (10 mg/L), up-regulation of miR-125a-5p depressed cell viability (Figure 3E), and promoted apoptosis in Ishikawa/PA and HEC-1A/PA cells (Figure 3F). Up-regulation of miR-125a-5p also significantly increased paclitaxel-induced cytotoxicity.

### MiR-125a-5p Mediated the Chemoresistance-Suppressive Effects of Knockdown of CDKN2B-AS in EC Cells

**Figure 4:**
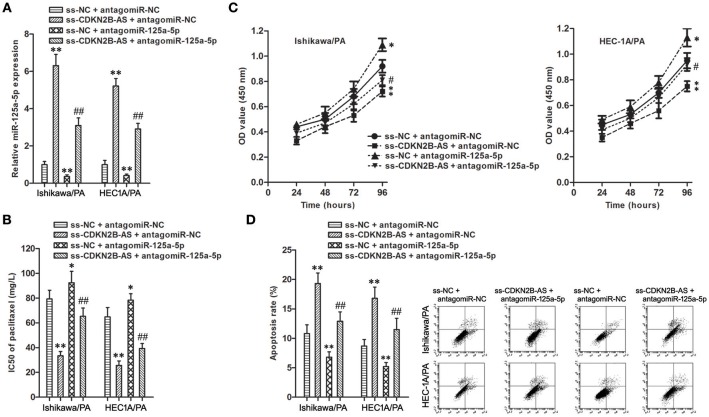
miR-125a-5p mediated the chemoresistance-suppressive effects of CDKN2B-AS knockdown in EC cells**. (A)** The expression of miR-125a-5p gene in Ishikawa/PA and HEC-1A/PA cells. **P < 0.01 vs. ss-NC + antagomiR-NC group; ## *P* < 0.01 vs. ss-NC + antagomiR-125a-5p group. **(B)** The IC50 of paclitaxel in Ishikawa/PA and HEC-1A/PA cells. **P < 0.01 agomiR-NC group. *P < 0.05 vs. ss-NC + antagomiR-NC group; **P < 0.01 vs. ss-NC + antagomiR-NC group; ##*P* < 0.01 vs. ss-NC + antagomiR-125a-5p group. **(C)** The cell viability of Ishikawa/PA and HEC-1A/PA cells under treating with 10 mg/L paclitaxel. **P < 0.01 vs. agomiR-NC group. *P < 0.05 vs. ss-NC + antagomiR-NC group; **P < 0.01 vs. ss-NC + antagomiR-NC group; #*P* < 0.05 vs. ss-NC + antagomiR-125a-5p group. **(D)** The cell apoptosis of Ishikawa/PA and HEC-1A/PA cells under treating with 10 mg/L paclitaxel. **P < 0.01 vs. ss-NC + antagomiR-NC group; ##*P* < 0.01 vs. ss-NC + antagomiR-125a-5p group.

To determine whether the chemoresistance-suppressive effects of CDKN2B-AS knockdown were mediated by miR-125a-5p, antagomiR-125a-5p was transfected into stable ss-CDKN2B-AS cells (Figure 4A). Co-transfection of ss-CDKN2B-AS with antagomiR-125a-5p showed the lower paclitaxel sensitivity compared with co-transfection of ss-CDKN2B-AS with antagomiR-NC, and transfection with antagomiR-125a-5p rescued the inhibitory effects of ss-CDKN2B-AS on paclitaxel resistance (Figures 4B–D). Based on the above-mentioned results, we confirmed that miR-125a-5p mediates suppressive effects of CDKN2B-AS knockdown on paclitaxel resistance in EC cells.

### Bcl2 and MRP4 Are Target Genes of miR-125a-5p in EC Cells

**Figure 5:**
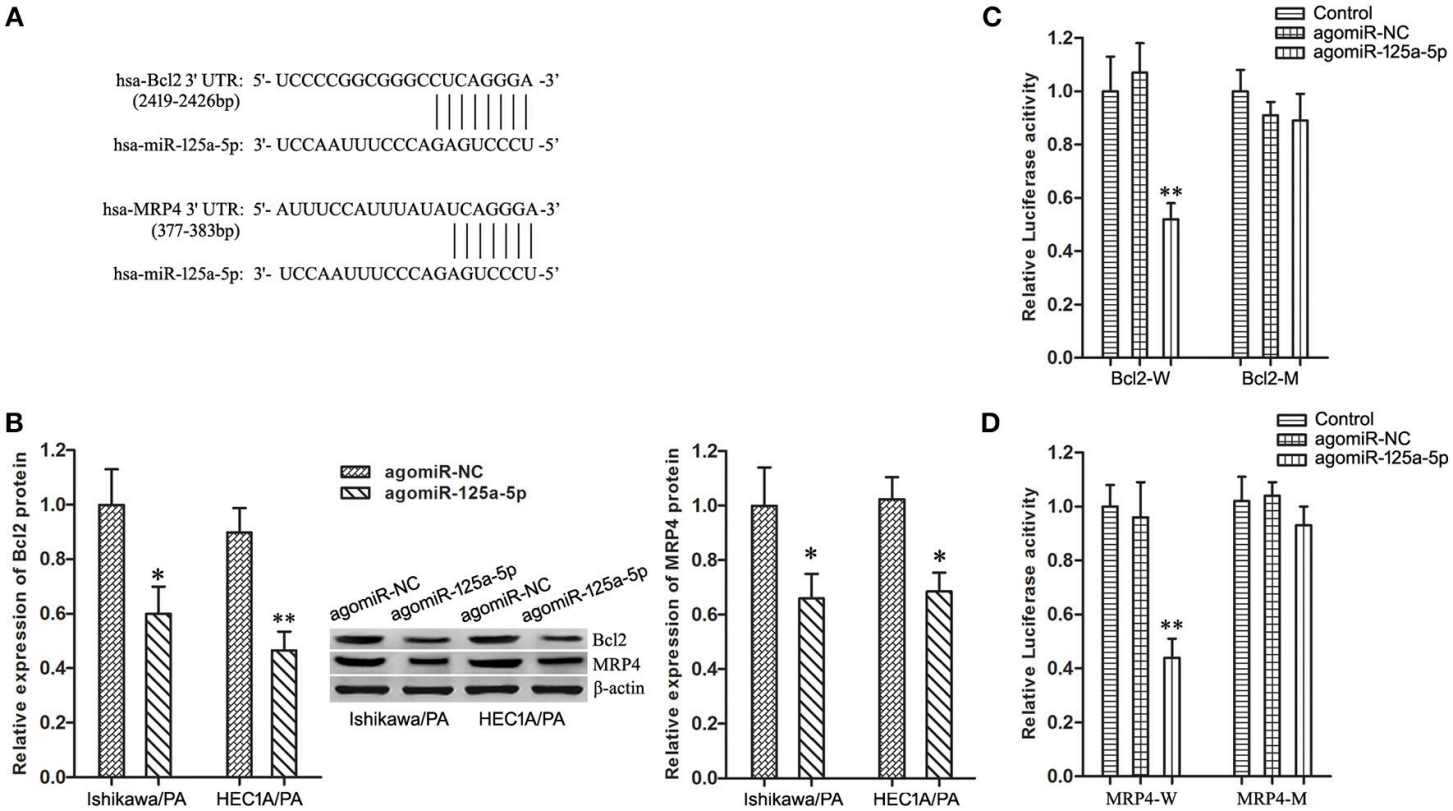
Bcl2 and MRP4 are target genes of miR-125a-5p in EC cells**. (A)** The predicted miR-125a-5p binding site in the 3′UTR of Bcl2 and MRP4 mRNA. Short vertical lines indicated complementary nucleotides. **(B)** The expression of Bcl2 and MRP4 protein in Ishikawa/PA and HEC-1A/PA cells. *P < 0.05 vs. agomiR-NC group. **P < 0.01 vs. agomiR-NC group. **(C)** The relative luciferase reporter assay of HEK 293T cells co-transfected with Bcl2-W or Bcl2-M and agomiR-125a-5p or agomiR-NC. **P < 0.01 vs. Bcl2-W + agomiR-NC group. **(D)** The relative luciferase reporter assay of HEK 293T cells co-transfected with MRP4-W or MRP4-M and agomiR-125a-5p or agomiR-NC. **P < 0.01 vs. MRP4-W + agomiR-NC group.

Based on information obtained from the online database TargetScan 7.1, Bcl2 and MRP4 might be targets of miR-125a-5 (Figure 5A). Firstly, up-regulation of miR-125a-5p remarkably depressed the expression level of Bcl2 and MRP4 in Ishikawa/PA and HEC-1A/PA cells (Figure 5B). Then, the specific binding sites of miR-125a-5p in the 3′UTR of Bcl2 and MRP4 were confirmed by luciferase reporter assay. In the Bcl2-W group, the luciferase activity of co-transfection with agomiR-125a-5p was inhibited, while no significant change was observed in their NC group; in the Bcl2-M group, the luciferase activity remained unchanged (Figure 5C). In addition, similar results were observed in MRP4 (Figure 5D). The results confirmed our prediction that Bcl2 and MRP4 are targets of miR-125a-5p in EC cells.

### Enhancement of Bcl2 and MRP4 Partially Reversed the Suppressive Effects of Up-Regulation of miR-125a-5p on Paclitaxel Resistance in EC Cells

**Figure 6:**
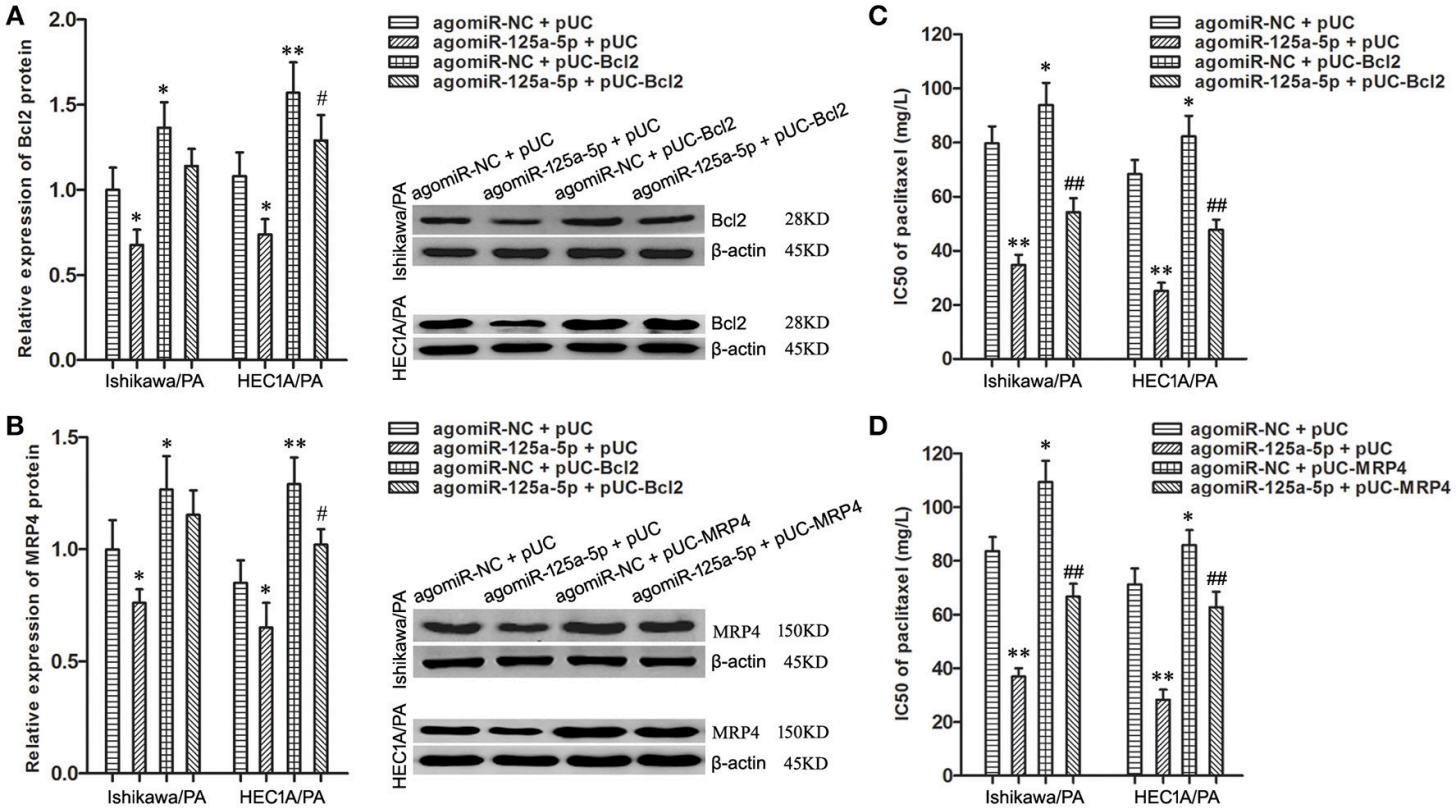
Enhancement of Bcl2 and MRP4 partially reversed the suppressive effects of up-regulation of miR-125a-5p on paclitaxel resistance in EC cells. **(A)** The expression of Bcl2 protein in Ishikawa/PA and HEC-1A/PA cells. *P < 0.05 vs. agomiR-NC + pUC group; **P < 0.01 vs. agomiR-NC + pUC group; #*P* < 0.05 vs. agomiR-125a-5p + pUC group. **(B)** The expression of MRP4 protein in Ishikawa/PA and HEC-1A/PA cells. *P < 0.05 vs. agomiR-NC + pUC group; **P < 0.01 vs. agomiR-NC + pUC group; #*P* < 0.05 vs. agomiR-125a-5p + pUC group. **(C,D)**: The IC50 of paclitaxel in Ishikawa/PA and HEC-1A/PA cells. *P < 0.05 vs. agomiR-NC + pUC group; **P < 0.01 vs. agomiR-NC + pUC group; ##*P* < 0.01 vs. agomiR-125a-5p + pUC group.

To uncover whether Bcl2 and MRP4 could reverse the chemoresistance-suppressive effects of up-regulation of miR-125a-5p in EC cells, the cells were co-transfected with agomiR-125a-5p, as well as pUC-Bcl2 or pUC-MRP4. Western blot analysis demonstrated that pUC-Bcl2 could up-regulate the expression level of Bcl2 in agomiR-125a-5p + pUC-Bcl2 group compared with agomiR-125a-5p + pUC-NC group (Figure 6A). In addition, similar results were achieved with pUC-MRP4 transfection (Figure 6B).

Drug sensitivity assay showed that up-regulation of miR-125a-5p sensitized Ishikawa/PA and HEC-1A/PA cells to paclitaxel, and over-expression of Bcl2 promoted the paclitaxel resistance and notably decreased miR-125a-5p-triggered increasing in paclitaxel sensitivity (Figure 6C). Similarly, over-expression of MRP4 also partially rescued the promotive effects of miR-125a-5p's on paclitaxel sensitivity (Figure 6D). Enhancement of Bcl2 and MRP4 could partially reverse the suppressive effects of up-regulation of miR-125a-5p on paclitaxel resistance in EC cells. These results illustrated that miR-125a-5p suppressed paclitaxel resistance by targeted silencing Bcl2 and MRP4 in EC cells.

## Discussion

Recent studies reported that lncRNAs were involved in the formation and maintenance of chemotherapy being resistance in almost all malignant tumors, including EC. For example, KCNQ1 Opposite Strand/Antisense Transcript 1 (KCNQ1OT1) was closely associated with insensitivity of lung adenocarcinoma, and also knockdown of KCNQ1OT1 depressed the expression level of Multidrug Resistance Protein 1 (MDR1) and the paclitaxel resistance (28). Urothelial carcinoma associated 1 (UCA1) is an independent prognostic biomarker, which contributed to adriamycin resistance in gastric cancer (29).

Paclitaxel is a broad-spectrum antitumor drug that is used in chemotherapy for a variety of tumors, including EC. Paclitaxel is isolated from the bark of the Pacific yew tree, and it can bind to tubulin and inhibit the disassembly of microtubules, thereby causing the obstacle of cell division and the death of tumor cells. However, paclitaxel resistance is a major factor associated with treatment failure.

In this study, CDKN2B-AS gene was highly expressed in EC tissues and cell lines, and its over-expression was positively associated with high pathological grade of EC, which suggested that CDKN2B-AS gene was involved in the progress of EC. Furthermore, the over-expression of CDKN2B-AS was associated with the low sensitivity to paclitaxel of EC patients, which preliminarily confirmed that CDKN2B-AS participated in the genesis of chemotherapy resistance in EC. Lan et al. reported that knockdown of CDKN2B-AS inhibited the development of multidrug resistance in gastric cancer cells to paclitaxel or 5-fluorouracil (30). CDKN2B-AS also contributed to paclitaxel resistance of lung adenocarcinoma A549 cells (31).

To verify the roles of CDKN2B-AS on paclitaxel resistance, the impacts of CDKN2B-AS on paclitaxel resistance in EC were examined by a series of loss-of-function assays. Knockdown of CDKN2B-AS decreased IC50 of paclitaxel in Ishikawa/PA and HEC-1A/PA cells, and also promoted the cytotoxicity induced by paclitaxel, which showed that knockdown of CDKN2B-AS increased paclitaxel sensitivity in EC cells.

Recently, lncRNAs were proposed to act as miRNA sponges or competitive endogenous RNAs (ceRNAs), forming extensive regulatory networks, thereby negatively regulating miRNA expression (32). For instance, LINC00161 sensitized the osteosarcoma cells to cisplatin through sponging miR-645 (33); lncRNA AC023115.3 acted as a ceRNA for miR-26a to inhibit cisplatin resistance of glioma cells (34). Using online bioinformatics databases, we predicted a negative regulatory relationship between CDKN2B-AS and miR-125a-5p. Furthermore, non-coding RNAs can ordinarily form ribonucleoprotein (RNP) complexes with their partner proteins to exert their functions and miRNAs assembling with Argonaute (Ago) family proteins into an effective complex called RISC, mediating silencing the target gene (35, 36).

A limited number of researches on miR-125a-5p and chemotherapy resistance were conducted. To date, only one literature reported that up-regulation of miR-125a-3p could advance docetaxel sensitivity of breast cancer cells through modulation of BRCA1 signaling (37). Little attention has been paid to the miR-125a-5p-associated chemotherapy effect in EC, especially to paclitaxel. Therefore, the correlation between the expression of miR-125a-5p and paclitaxel resistance in EC was here examined, and the results revealed that its low-expression was related to poor paclitaxel response of EC patients. Furthermore, enhanced miR-125a-5p decreased IC50 of paclitaxel in Ishikawa/PA and HEC-1A/PA cells, and promoted the paclitaxel-induced cytotoxicity, which showed that over-expression of miR-125a-5p could increase paclitaxel sensitivity in EC cells.

We attempted to explore whether the influence of CDKN2B-AS on paclitaxel resistance is mediated by miR-125a-5p in EC. In detail, the regulatory relationship between CDKN2B-AS and miR-125a-5p was confirmed based on the following results: (1) Knockdown of CDKN2B-AS significantly increased the expression level of miR-125a-5p in Ishikawa/PA and HEC-1A/PA cells; (2) RNA pull-down assay revealed CDKN2B-AS function via interaction with miR-125a-5p; (3) RIP experiment confirmed that CDKN2B-AS and miR-125a-5p were associated with Ago-2 protein in a RISC complex, and suggested that CDKN2B-AS decreased the expression level of miR-125a-5p in a RISC-dependent manner; (4) miR-125a-5p deficiency rescued the inhibitory effect of knockdown of CDKN2B-AS on paclitaxel resistance. Based on the above-mentioned achievements, we confirmed that miR-125a-5p mediates the suppressive effects of knockdown of CDKN2B-AS on paclitaxel resistance in EC cells.

As microRNAs play their roles through regulating their target genes, such as miR-16-1 and FUBP1 (38), we predicted that Bcl2 and MRP4 genes might be miR-125a-5p's target genes based on the online bioinformatics database. In addition, Tong et al. reported that miR-125a-5p could regulate cytobiological phenotypes of colon cancer via targeting Bcl2 (20). A series of following gain-of-function experiments, such as luciferase reporter assay and Western blotting, demonstrated that Bcl2 and MRP4 genes were the target genes of miR-125a-5p. To sum up, we speculate that miR-125a-5p might modulate the paclitaxel resistance of EC cells through targeted silencing Bcl2 and MRP4.

To verify this hypothesis, knockdown of Bcl2 and MRP4 by miR-125a-5p enhancement was rescued by transfection with pUC-Bcl2 and pUC-MRP4. The following experiments found that up-regulation of Bcl2 and MRP4 separately reversed the regulatory roles of miR-125a-5p on paclitaxel resistance in Ishikawa/PA and HEC-1A/PA cells. Besides, miR-125a-5p might promote cell apoptosis and reduce paclitaxel discharge through silencing targeted Bcl2 and MRP4, and then re-sensitize Ishikawa/PA and HEC-1A/PA cells to paclitaxel.

To sum up, CDKN2B-AS is specifically combined with miR-125a-5p and also down-regulates its expression level in EC, weakening the ability of miR-125a-5p to silence its target genes, and then up-regulates the expression of Bcl2 and MRP. Additionally, Bcl2 can inhibit apoptosis and promote cell survival from paclitaxel-induced cytotoxicity. Moreover, an overexpressed MRP4 pumps paclitaxel out of EC cells, and reduced intracellular paclitaxel concentration and paclitaxel-induced chemotherapy damage. Accordingly, CDKN2B-AS can desensitize EC cells into paclitaxel, and contribute to the formation of chemotherapy resistance through miR-125a-5p/Bcl2 and miR-125a-5p/MRP4 pathways.

In conclusion, high-expression of CDKN2B-AS is associated with a poor response to paclitaxel of EC patients, and knockdown of CDKN2B-AS inhibits paclitaxel resistance through miR-125a-5p-Bcl2/MRP4 pathway in EC. Our findings helped elucidate the underlying mechanism of chemotherapeutic resistance in EC patients.

## Author Contributions

LM and CS conceived and designed the project. CS and CA completed the experiments and acquired the data. CA and CC analyzed the data. All authors read and approved the final version of this manuscript.

### Conflict of Interest Statement

The authors declare that the research was conducted in the absence of any commercial or financial relationships that could be construed as a potential conflict of interest.
